# Referral patterns of stroke rehabilitation inpatients to a model system of outpatient services in Ontario, Canada: a 7-year retrospective analysis

**DOI:** 10.1186/s12913-019-4236-5

**Published:** 2019-06-20

**Authors:** Shannon Janzen, Magdalena Mirkowski, Amanda McIntyre, Swati Mehta, Jerome Iruthayarajah, Robert Teasell

**Affiliations:** 1 Parkwood Institute Research, Lawson Health Research Institute, Parkwood Institute, London, Ontario Canada; 2 Schulich School of Medicine & Dentistry, University of Western Ontario, London, Ontario Canada; 3 St. Joseph’s Health Care London, Physical Medicine and Rehabilitation, Parkwood Institute, London, Ontario Canada

**Keywords:** Stroke, Referral patterns, Care pathways, Outpatient rehabilitation, Inpatient rehabilitation, Outpatient services

## Abstract

**Background:**

While several studies have tracked the care paths of patients in the early phases of stroke recovery, studies examining the transition from inpatient to outpatient rehabilitation are lacking. Examining this transition allows for improved understanding and refinement of the process whereby patients are referred and admitted to programs. The objective of this study was to examine the referral patterns of stroke rehabilitation inpatients to outpatient stroke therapy services, their demographics, and clinical profile.

**Methods:**

This study examined patients who: (1) were admitted to an inpatient stroke rehabilitation unit between January 1, 2009 and March 1, 2016, (2) had a stroke diagnosis, (3) had an inpatient length of stay of > 1 day, and (4) lived within the geographical boundaries of the South West Local Health Integration Network which allowed them access to both hospital-based and home-based stroke rehabilitation outpatient programs. Patient data was collected from the National Rehabilitation Reporting System, as well as three hospital outpatient administrative databases. These databases were cross-referenced to determine each patient’s pathway. Those referred to an outpatient therapy program, and those who attended the outpatient programs, were compared to those who were not, and did not, respectively.

**Results:**

1497 inpatients were included in the analysis. Upon discharge, 1037 (69.3%) of patients had an outpatient clinic, follow-up appointment scheduled; of those, 902 (87.0%) patients attended at least one outpatient clinic visit. 891 (59.5%) were referred to one of the interdisciplinary outpatient stroke rehabilitation programs; of those, an outpatient therapy program was attended by 80.9% of patients (*n* = 721). Of those receiving outpatient therapy services, the number of patients attending the in-hospital versus home-based program were equal, 360 and 361 individuals, respectively.

**Conclusion:**

This study allows for a better understanding of the transition between inpatient and outpatient stroke care. There is a paucity of this type of information in stroke rehabilitation literature to date. This study acts as a starting point in improving rehabilitation planning across the continuum of care.

## Background

In Canada, stroke is a leading cause of disability and the third leading cause of death [1]; more than 50,000 Canadians experience a stroke every year [2]. Among stroke survivors, the estimated economic burden to the acute care health care system alone exceeds $1.1 billion [3]. To date, the scientific literature has provided strong evidence for the effectiveness of several acute and rehabilitation interventions for individuals post stroke [4]. Despite these studies, there is limited research examining longitudinal models of stroke care.

The Canadian Best Practice Recommendations for Stroke Care currently represent an evidenced-based approach to effective stroke care, and recommend “a coordinated and integrated approach to stroke prevention, treatment, rehabilitation and community reintegration in every province and territory in Canada” [5]. In Canada, the continuum of stroke care commonly includes four levels of stroke care: (1) hyperacute stroke management; (2) acute stroke management; (3) stroke rehabilitation; and (4) community reintegration. It has been acknowledged that an integrated, coordinated continuum of care for stroke patients leads to better patient outcomes [6]; however, each level of care accessed by patients and health providers often functions independently which can hinder movement of patients or flow of information transferred between centres. While there has been a sizable increase in the use of technology-enabled approaches in the management of stroke care over the last decade, there is still a noticeable lack of a centralized tracking, monitoring, and care coordination system for stroke patients, particularly between inpatient, outpatient, and community rehabilitation. While the importance of a continuum of stroke care is widely acknowledged, there is limited information about how the continuum actually works.

Despite acute and inpatient stroke services having regulated administrative datasets (i.e., the National Rehabilitation Reporting System), no such mandate applies to stroke outpatient rehabilitation in Ontario, Canada. Consequently, a survey of stroke facilities in Ontario, Canada found that only 38.1% of respondents could provide basic data on their outpatient programs (e.g., number of treatments and patients treated) [7]. In Ontario there are 14 Local Health Integration Networks (LHIN) which are responsible for the regional administration of health care services which include public and private hospitals, long-term care homes, and several community support services [8]. It was estimated that more than 500 individuals will need access to outpatient or community-based stroke rehabilitation annually in the South West LHIN (SW LHIN) [9], a regional health care network responsible for almost one million people in Southwestern Ontario. Of concern, this projection was based on several assumptions (i.e., survival rates). Since there was an extreme lack of outpatient data available for the authors to evaluate, it is expected that this figure represents a significant underestimation of the number of patients receiving and requiring services.

Recent studies have shown that the most cost-effective method of providing rehabilitation is dependent on both the services available and individual patient characteristics [10]. While it is known which outpatient services exist, to our knowledge, there has been no study that has systematically tracked stroke patients being discharged from an inpatient setting and identified follow-up services within the Canadian health care system. Additionally, no study has assessed differences in individual demographic or clinical variables between those who receive or do not receive follow-up services. Examining the transition between inpatient and outpatient stroke rehabilitation allows for improved understanding and refinement of the process whereby patients are referred and admitted to programs. Understanding current practices is necessary and a first step in developing recommendations for streamlining the care continuum, closing gaps to ensure patients are not left behind, and ultimately optimizing health care delivery. Individuals in the SW LHIN have access to both hospital-based and home-based interdisciplinary specialized stroke rehabilitation care. Therefore, the objective of this study was to examine the referral patterns of stroke rehabilitation inpatients to outpatient stroke therapy services, their demographics, and clinical profile.

## Methods

### Ethics

This study was approved by Western University’s Health Sciences Research Ethics Board (HSREB 105215).

### Design

This study was a retrospective chart review of all patients admitted to stroke inpatient rehabilitation over a seven-year time period.

### Setting

Patients included in this study received inpatient specialized stroke rehabilitation at a stand-alone academic rehabilitation hospital within Southwestern Ontario. This facility is the largest of five centers that offer inpatient rehabilitation stroke care within the SW LHIN and is the sole rehabilitation centre for people in London, Ontario and immediate surroundings. Upon discharge, several outpatient services are available to patients by physician referral. Referrals may be made to 1) a physician- and nurse-led outpatient clinic, 2) a hospital-based interdisciplinary outpatient therapy program, and/or 3) a home-based interdisciplinary outpatient therapy program. The outpatient clinic provides follow-up visits where patients are assessed by a physiatrist and a nurse; subsequent referrals for therapy may be made as appropriate. While the physician signs off on the referral to outpatient services, the decision is made collectively by the rehabilitation team and physician, in conjunction with the patient. The hospital-based program is for individuals who require rehabilitation programs from at least one therapist, have neurological treatment needs that cannot be met by community services, have potential for functional/neurological improvement, are medically stable, and have reliable transportation. The home-based program has similar criteria; the individual must be a stroke survivor, medically stable, have potential for functional improvement, and require specialized stroke services. The home-based and hospital-based outpatient programs both offer the services of a physiotherapist, speech language pathologist, social worker, and occupational therapist. The home-based program also offers services by registered nurses, therapeutic recreation specialists, and rehabilitation therapists, who help implement the programs prescribed by the other health professionals.

### Data collection

Data for all inpatients were collected from the National Rehabilitation Reporting System. Inpatient data included: age, gender, date of stroke onset, inpatient admission and discharge dates, discharge living setting, rehabilitation client group (RCG), rehabilitation patient group (RPG), postal code, and admission and discharge total Functional Independence Measure (FIM) scores, as well as the FIM cognitive and motor sub-scores at both time points. The FIM is an 18-item measure that contains 5 cognitive and 13 motor items. Each item is scored on a 7-point scale based on the level of assistance required to complete activities of daily living [11]. Scores range from 18 to 126, with higher scores indicating greater functional independence. RCGs are health condition categorizations (17 categorizations); each patient has a single RCG that best describes their primary reason for admission to inpatient rehabilitation. RPGs are classifications of patient stroke severity which have been determined through an algorithm based on patient FIM scores (motor and cognitive) and age [12]. RPGs were then categorized into mild (1150–1160), moderate (1120–1140), and severe (1100–1110) strokes.

A list of all patients referred or attending the aforementioned outpatient clinic and home- or hospital-based outpatient therapy programs were collected from the corresponding administrative dataset. For the outpatient therapy programs, the following were collected: referral date, admission and discharge date to the program, and the total number of therapy visits provided to each patient. These databases were cross-referenced with the inpatient data to determine each patient’s pathway. The outpatient therapy dates were used to determine which program (home or hospital), if any, the patients attended first. Additional services accessed outside of these three programs were not examined.

### Study inclusion

Patients meeting the following study inclusion were included in the analysis: (1) admitted to the stroke rehabilitation unit between January 1, 2009 and March 1, 2016, (2) had a stroke RCG, (3) had an inpatient length of stay of > 1 day, and (4) lived within the geographical boundaries of the SW LHIN (determined by postal code) which allowed them access to both hospital-based and home-based stroke rehabilitation outpatient programs. Individuals outside the SW LHIN would not necessarily have access to the home-based interdisciplinary outpatient program and were therefore excluded. For each patient, only the first outpatient program (home or hospital-based) attended was included for analysis.

### Data analysis

Descriptive statistics were calculated for all variables collected. Means with standard deviations were used to describe continuous variables, and counts and percentages were used for categorical data. Length of stay on the inpatient unit, FIM gains (motor, cognitive, total), and wait time from stroke onset to inpatient admission were calculated based on the National Rehabilitation Reporting System data provided. Distance to the hospital was determined as the shortest driving route between the patient’s area (determined by postal code) and the hospital address. Those referred to an outpatient therapy program were compared to those who were not. Post referral, those who received outpatient therapy services were compared to those who did not. For both analyses, further comparisons based on discharge setting (e.g., home or other institution) were performed, where other institution refers to long-term care, residential care, re-admissions to the hospital, and unknown. Between group differences were determined using independent t-tests and chi-square tests, as appropriate. Statistical significance was set at *p* < 0.05. All data were organized and analyzed using SPSS version 23.0.

## Results

### Demographics

Of the 1766 patients admitted to the stroke inpatient rehabilitation unit, 1497 were included in the analysis. Reasons for exclusion were: non-stroke RCG (*n* = 169), length of stay ≤1 day (*n* = 6), and lived outside the SW LHIN (*n* = 94). Of the study population, 32 patients had two separate strokes and two patients had three separate strokes. Because each stroke was followed by a new inpatient rehabilitation stay, each case was considered unique. The sample had a mean age of 69.1 ± 14.3 years (Table 1). The median time between stroke onset and admission to the inpatient unit was 13 days. The mean inpatient length of stay was 35.7 ± 29.5 days (median = 29.0 days), with a mean FIM gain of 21.7 ± 15.5.

**Table 1:** Inpatient Demographics

Demographic Characteristics	Stroke Inpatients *N* = 1497
Mean Age, years	69.1 ± 14.3
Gender (%)	
Male	781 (52.2)
Female	683 (45.6)
Unknown	33 (2.2)
Stroke Severity (%)	
Mild	290 (19.4)
Moderate	667 (44.6)
Severe	540 (36.1)
Rehabilitation Client Group (%)	
Stroke Left Body	607 (40.5)
Stroke Right Body	678 (45.3)
Stroke Bilateral	65 (4.3)
Stroke No Paresis	73 (4.9)
Other Stroke	74 (4.9)
Mean Admission FIM	
Motor	51.5 ± 21.2
Cognitive	25.5 ± 6.5
Total	77.0 ± 24.4
Mean Discharge FIM	
Motor	70.7 ± 19.7
Cognitive	28.4 ± 5.6
Total	99.1 ± 22.9
Mean FIM Gain	
Motor	18.9 ± 14.0
Cognitive	2.7 ± 3.6
Total	21.7 ± 15.5
Mean Inpatient Length of Stay, days	35.7 ± 29.5

Mean ± Standard deviation, FIM = Functional Independence Measure

### Follow-up in outpatient clinic

Upon discharge from inpatient rehabilitation, 1037 (69.3%) patients had an outpatient clinic follow-up appointment scheduled; of which, 902 (87.0%) attended at least one outpatient clinic visit.

### Who was referred to outpatient stroke rehabilitation?

**Fig. 1:**
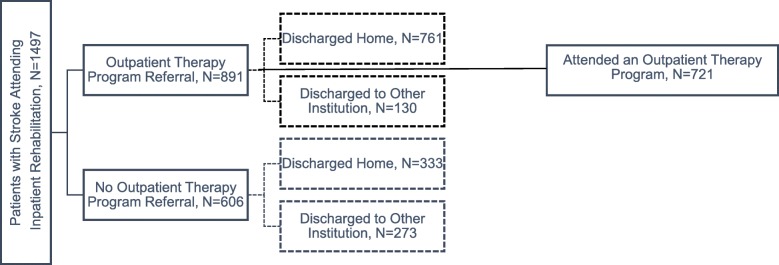
Outpatient referrals following inpatient rehabilitation

Of the 1497 stroke rehabilitation inpatients discharged, 891 (59.5%) were referred to one of the outpatient stroke rehabilitation programs (Fig. 1). When comparing those who received a referral and those who did not, the groups differed significantly in terms of stroke severity (*p* = 0.005). Those who received referrals were significantly younger, had shorter inpatient lengths of stay, lived closer to the hospital, and had higher motor FIM scores at admission, and higher FIM scores (total, cognitive, and motor) at discharge (*p* < .05) than those who did not receive a referral.

Among those who received a referral (*n* = 891, 59.5%), the majority (85.4%) were discharged home as opposed to another institution. Of those who were not referred, the proportion discharged home or to another institution were more similar, 55.0% (mean discharge FIM of 108.7) and 45.0% (mean discharge FIM of 73.6), respectively. The patients who were referred and not referred to outpatient therapy but discharged to another institution appeared similar with the exception that those referred had higher total and motor FIM scores at discharge, and were living closer to the hospital. A comparison of those who were and were not referred based on their discharge setting (e.g., home or other institution) is provided in Table 2.

**Table 2:** Comparison of those referred and not referred based on discharge destination

Demographic Characteristics	Outpatient Therapy Program Referral, N = 891		No Outpatient Therapy Program Referral, *N* = 606		Home p-value	Other *p*-value
	Discharged Home *N* = 761	Discharged to Other Institution *N* = 130	Discharged Home *N* = 333	Discharged to Other Institution *N* = 273		
Mean Age, years	66.7 ± 13.4	73.6 ± 12.5	67.9 ± 15.9	75.6 ± 13.9	0.233	0.220
Gender (%)						
Male	423 (55.6)	68 (52.3)	167 (50.2)	123 (45.1)	0.399	0.332
Female	337 (44.3)	61 (46.9)	149 (44.7)	136 (49.8)		
Unknown	1 (0.1)	1 (0.8)	17 (5.1)	14 (5.1)		
Stroke Severity (%)						
Mild	164 (21.6)	6 (4.6)	105 (31.5)	15 (5.5)	< 0.001	0.379
Moderate	379 (49.8)	47 (36.2)	161 (48.3)	80 (29.3)		
Severe	218 (28.6)	77 (59.2)	67 (20.1)	178 (65.2)		
Rehabilitation Client Group (%)						
Stroke Left Body	289 (38.0)	50 (38.5)	131 (39.3)	137 (50.2)	0.088	0.104
Stroke Right Body	357 (46.9)	68 (52.3)	147 (44.1)	106 (38.8)		
Stroke Bilateral	40 (5.3)	3 (2.3)	10 (3.0)	12 (4.4)		
Stroke No Paresis	35 (4.6)	3 (2.3)	27 (8.1)	8 (2.9)		
Other Stroke	40 (5.3)	6 (4.6)	18 (5.4)	10 (3.7)		
Mean Admission FIM						
Motor	55.2 ± 20.2	39.0 ± 17.8	60.3 ± 19.2	36.3 ± 16.9	< 0.001	0.143
Cognitive	26.1 ± 6.1	22.9 ± 7.0	27.5 ± 5.7	22.8 ± 6.8	< 0.001	0.926
Total	81.3 ± 22.7	61.9 ± 20.8	87.8 ± 22.2	59.1 ± 20.3	< 0.001	0.208
Mean Discharge FIM						
Motor	76.6 ± 14.4	56.5 ± 20.5	78.6 ± 13.3	48.6 ± 21.3	0.026	0.001
Cognitive	29.1 ± 4.9	25.3 ± 6.4	30.2 ± 4.6	25.0 ± 6.5	0.001	0.624
Total	105.7 ± 16.7	81.8 ± 23.7	108.7 ± 16.2	73.6 ± 24.2	0.006	0.002
Mean Inpatient Length of Stay, days	30.4 ± 16.2	48.8 ± 30.3	27.2 ± 16.7	54.3 ± 51.9	0.003	0.177
Mean Distance to Hospital, km	17.4 ± 18.2	20.0 ± 19.4	34.0 ± 39.8	28.0 ± 35.6	< 0.001	0.004

*p*-values are provided for between group differences, Mean ± Standard deviation, FIM = Functional Independence Measure

### Among outpatients, who received treatment?

Of the 891 referrals, an outpatient therapy program was attended by 80.9% of patients (*n* = 721). Those who attended the outpatient therapy programs were significantly younger (66.8 ± 13.7 vs. 71.5 ± 11.8, *p* < 0.001), and had higher total FIM scores at discharge (103.6 ± 19.2 vs 97.4 ± 20.7, p < 0.001), as well as higher motor (74.8 ± 16.3 vs 69.7 ± 18.4, *p* = 0.001) and cognitive (28.8 ± 5.4 vs 27.7 ± 5.0, *p* = 0.019) FIM subscale scores at discharge. There was no significant difference in stroke severity between individuals who did and did not attend therapy (*p* = .065). When examining those discharged to other institutions who did and did not attend therapy, there were no significant differences between groups (Table 3). When examining those discharged home, those who actually attended therapy were significantly younger and had higher inpatient FIM discharge scores.

**Table 3:** Post referral, patients who received outpatient therapy services versus those who did not based on discharge setting

	Outpatient Therapy Services Received, N = 721		No Outpatient Therapy Services Received, *N* = 170		Between group - Home	Between group - Other
Demographic Characteristics	Discharged Home *N* = 626	Discharged to Other Institution *N* = 95	Discharged Home *N* = 135	Discharged to Other Institution *N* = 35		
Mean Age, years	65.8 ± 13.5	73.6 ± 13.1	70.9 ± 12.0	73.8 ± 10.9	< 0.001	0.917
Gender (%)						
Male	352 (56.2)	47 (49.5)	71 (52.6)	21 (60.0)	0.429	0.312
Female	273 (43.6)	47 (49.5)	64 (47.4)	14 (40.0)		
Unknown	1 (0.2)	1 (1.1)	0 (0)	0 (0)		
Stroke Severity (%)						
Mild	141 (22.5)	6 (6.3)	23 (17.0)	0 (0)	0.361	0.201
Moderate	309 (49.4)	36 (37.9)	70 (51.9)	11 (31.4)		
Severe	176 (28.1)	53 (55.8)	42 (31.1)	24 (68.6)		
Rehabilitation Client Group (%)						
Stroke Left Body	238 (38.0)	30 (31.6)	51 (37.8)	20 (57.1)	0.240	0.082
Stroke Right Body	299 (47.8)	54 (56.8)	58 (43.0)	14 (40.0)		
Stroke Bilateral	28 (4.5)	3 (3.2)	12 (8.9)	0 (0)		
Stroke No Paresis	27 (4.3)	3 (3.2)	8 (5.9)	0 (0)		
Other Stroke	34 (5.4)	5 (5.3)	6 (4.4)	1 (2.9)		
Mean Admission FIM						
Motor	55.4 ± 20.4	40.2 ± 17.3	54.5 ± 19.6	35.8 ± 18.9	0.634	0.217
Cognitive	26.2 ± 6.2	22.9 ± 7.3	25.3 ± 5.7	22.7 ± 6.3	0.124	0.890
Total	81.6 ± 22.9	63.1 ± 20.7	79.8 ± 21.8	58.5 ± 21.0	0.394	0.271
Mean Discharge FIM						
Motor	77.3 ± 14.1	57.6 ± 19.6	73.4 ± 15.0	53.5 ± 23.2	0.004	0.342
Cognitive	29.3 ± 5.0	25.2 ± 6.5	28.2 ± 4.6	25.7 ± 6.1	0.017	0.731
Total	106.6 ± 16.5	82.8 ± 23.0	101.6 ± 16.8	79.1 ± 25.9	0.002	0.465
Mean Inpatient Length of Stay, days	30.5 ± 16.6	48.4 ± 28.1	30.2 ± 14.3	49.7 ± 36.1	0.872	0.838
Mean Distance to Hospital, km	17.4 ± 18.5	18.9 ± 18.1	17.1 ± 16.7	23.0 ± 22.6	0.849	0.342

Mean ± Standard deviation, FIM = Functional Independence Measure

### Among outpatients, where did they receive treatment?

Of those receiving outpatient therapy services, the number of patients attending the in-hospital versus home-based program were equal, 360 and 361 individuals, respectively. Patients receiving outpatient therapy services received on average 32.2 ± 26.2 therapy visits.

## Discussion

While outpatient programs are evaluated in the literature, there is a lack of tracking patients across the stroke continuum. Another Ontario based study examined care trajectories for first ever stroke/transient ischemic attack patients and found that 17.7% of patients followed a pathway of emergency department, inpatient stay, and then inpatient rehabilitation [13]; however, there is a lack of information regarding the next care transition to outpatient rehabilitation. Our study examined the referral patterns of 1497 patients discharged from a stroke inpatient rehabilitation unit to subsequent outpatient stroke therapy services, as well as associated demographics and clinical profiles. This study is unique in that it examines clinical practices within the infrastructure of a model stroke system. In accordance with the Canadian Best Practice Recommendations for Stroke Care [14], stroke survivors who have ongoing rehabilitation needs should have access to specialized outpatient services post discharge, either in-home or at a facility, based on individual needs. The current SW LHIN system offers home- and hospital-based outpatient therapy programs which are comparable in the intensity and type of therapy provided, and both of which have demonstrated therapeutic benefit [15, 16].

The results of this study indicate that, although extensive outpatient services are available, not all stroke patients are referred, with the rate being approximately 60%. Given the lack of research on outpatient rehabilitation, it is unknown whether this is reflective of practices in other parts of Canada or if it is appropriate. An American survey of 21 states and the District of Columbia found that 30.7% of individuals with stroke received outpatient rehabilitation; however, this survey did not specifically mention inpatient rehabilitation and had several limitations [17]. Further, studies have examined the referral patterns of patients from post-acute stroke rehabilitation within an integrated health system (including inpatient rehabilitation facilities, skilled nursing facilities, home health care, and outpatient clinics) [18–20]. Given that the health care systems studied in these articles differ from that used in Canada, comparisons are challenging. Our data suggests that those being referred to outpatient stroke therapy from an inpatient stroke rehabilitation unit were younger, had a shorter inpatient length of stay, lived closer to the hospital, and had higher FIM scores at admission and discharge. Upon further examination of those who were not referred, 333 were discharged home and 273 were sent to a form of institutional care. These groups represent two extremes of a continuum; at one end of the extreme, those who went home were discharged with a mean total FIM score of 108.7 and a mean age of 67.9 years. At the other extreme, those discharged to an institution had a mean total FIM score of 73.6 and a mean age of 75.6 years. These groups of patients support the notion that patients who are functioning very well, or too poorly to benefit from continued rehabilitation, tend to not be referred for follow-up stroke rehabilitation therapy services.

When examining patients who did receive a referral, based on their discharge destination, it appears that a key determinant was their discharge FIM scores; this alludes to the fact that there may be a range of scores which are deemed acceptable for referring a patient. This is consistent with research showing stroke severity and FIM scores as key predictors of discharge destination [21, 22]. A systematic review of 18 studies examining predictors to institutional long-term care after hospitalization for stroke (acute or rehabilitation hospitals) found that older age, hemorrhagic stroke, having comorbidities or complications during their stay, greater stroke severity, and a lack of social support were associated with discharge to long-term care [21]. Further, from a sample of 148,367 individuals, admission FIM motor scores were shown to have the strongest correlation with being discharged home [22]. Thus, level of functional ability appears to not only impact discharge destination following hospitalization, but also decision-making regarding the need for continued rehabilitation after stroke.

A unique finding of this study is that not all patients who were referred to outpatient stroke therapy actually received these services; approximately 81% of patients attended an outpatient therapy program once referred. Patients not receiving therapy tended to be older and had significantly lower FIM scores upon discharge from inpatient rehabilitation. Upon closer examination, there were no demographic or clinical differences between patients discharged to institutional care, who did and did not receive therapy, which may indicate that the receipt of therapy once referred may be dependent on other factors. It is unknown from the current study whether patients chose to decline services, or otherwise encountered barriers to access. Johnson et al. [23] examined factors that influenced post-hospital discharge placement by an interdisciplinary rehabilitation team and, among others, found patient/family involvement in the decision-making process to be an important factor affecting favourable placement outcomes. It is unknown whether similar practices were used in this study and if patients were part of the decision-making team; however, the presence or absence of patient involvement may have a direct impact on a patient’s willingness to comply with the treatment and feasibility of attending the program. Further, research has shown that older individuals with severe stroke are less likely to have a caregiver compared to younger stroke patients [24]; this may create transportation issues, limiting their ability to get to a hospital-based program. However, it is difficult to estimate the generalizability of this assumption to our study sample, given that this study did not explore which program patients who did not receive therapy had been referred to originally. Additional barriers to engaging in stroke rehabilitation, from a patient perspective, may include inadequate discharge planning processes which negatively impact patient preparedness to engage in post-discharge rehabilitation, as well as a lack of communication, continuity, and coordination when transferred from one care setting to another [25]. Thus, the exploration of stroke survivors’ experiences specific to outpatient rehabilitation may assist with our understanding of realistic accessibility issues, if present, faced by this clinical population.

When patients attended an outpatient therapy program, it appeared the split between home and hospital was even. Similarly, a study found that when patients being discharged from an acute care hospital were advised about the most suitable rehabilitation setting from a geriatrician, then asked to decide whether to receive rehabilitation in an institution or at home, 52.4% of patients chose an institution and 47.6% home [26]. A significantly greater number of women, and individuals who were independent or partially independent in activities of daily living chose rehabilitation at home [26]. However, a study looking at 54 individuals who received inpatient rehabilitation, recruited from five health care systems in Texas, found that 84% of rehabilitative therapy was provided in an outpatient facility, opposed to in the home, in the first year post stroke [27]. A study of cardiac rehabilitation patients found that travel time/distance, level of supervision, perceived health benefits, the social aspects of the program, and the available facilities all contributed to their decision in where to receive therapy (home versus hospital) [28].

Our study raises questions regarding the referral process to, and receipt of, stroke outpatient rehabilitation services, and highlights gaps within our current system. It remains to be known whether the appropriate percentage of inpatients are receiving referrals to outpatient stroke rehabilitation. Research has suggested that access to rehabilitation services may be limited by program eligibility criteria, which outweighs demand [29]; as such, a less than ideal number of patients may be being referred to outpatient stroke therapy services. Further, questions remain regarding why some patients do not attend outpatient therapy once referred; regardless, the results of this study highlight a mismatch within our system that requires clarification in terms of whether this stems from a provider (i.e., ill-fitting referrals) or patient (i.e., declining services due to personal choice) standpoint.

This study has several limitations, one being that all data was accessed retrospectively and originally collected for administrative purposes; therefore, we are limited by the accuracy and completeness of the data collected. Further, while there was some overlap in the variables collected in the administrative datasets, this was not the case for other variables, such as why outpatient services were not attended post referral, limiting what information could be accessed for this study. Future studies should investigate reasons for not attending therapy, as well as potential associations with type of outpatient program. Further, to the best of our knowledge, there has been no other study to date which has investigated trends in referrals to outpatient stroke rehabilitation services in Canada. Therefore, a comparison of referral patterns between different facilities is not feasible at present, and future studies are encouraged to examine this further. Additionally, examining the availability and timeliness of access to outpatient services in different model systems would be beneficial.

## Conclusions

Ultimately, our study findings reinforce the need for a mandated centralized administrative dataset for patients entering outpatient stroke rehabilitation services. Standardized data reporting would overcome the limitations of current documentation practices, assist in the identification of patients who are not receiving therapy due to factors other than personal choice, and improve the provision of rehabilitation services to ensure continuity of care.

## Data Availability

All relevant data are within the manuscript. The dataset analyzed during the current study is available from the corresponding author on reasonable request.

## Abbreviations

FIM: Functional Independence Measure
RCG: Rehabilitation Client Group
RPG: Rehabilitation Patient Group
SW LHIN: South West Local Health Integration Network

## References

[1] Statistics Canada. Table 102-0561 - Leading causes of death, total population, by age group and sex, Canada, annual, CANSIM (database). 2017. https://www150.statcan.gc.ca/t1/tbl1/en/tv.action?pid=1310039401. Accessed 7 July 2017.

[2] Heart and Stroke Foundation. Together against a rising tide: Advancing stroke systems of care. Heart Stroke Foundation. 2014. https://www.strokebestpractices.ca/-/media/1-stroke-best-practices/quality/english/strokereport2014v2.ashx

[3] Mittmann N, Seung SJ, Hill MD (2012). Impact of disability status on ischemic stroke costs in Canada in the first year. Can J Neurol Sci..

[4] McIntyre A, Richardson M, Janzen S, Hussein N, Teasell R (2014). The evolution of stroke rehabilitation randomized controlled trials. Int J Stroke.

[5] Lindsay MP, Gubitz G, Bayley M, Hill MD, Phillips S, Smith EE (2014). Canadian Stroke Best Practice Recommendations Overview and Methodology.

[6] Cameron JI, Tsoi C, Marsella A (2008). Optimizing stroke systems of care by enhancing transitions across care environments. Stroke..

[7] Meyer M, Pereira S, McClure A (2011). An economic model for stroke rehabilitation in Ontario: mapping resource availability and patients needs.

[8] South West LHIN (2014). About our LHIN. Retrieved from http://www.southwestlhin.on.ca/aboutus.aspx. Accessed 21 May 2019.

[9] Meyer M, McClure A, O'Callaghan C (2013). Regional economic overview - South West LHIN.

[10] Brusco NK, Taylor NF, Watts JJ, Shields N (2014). Economic evaluation of adult rehabilitation: a systematic review and meta-analysis of randomized controlled trials in a variety of settings. Arch Phys Med Rehabil.

[11] Keith RA, Granger CV, Hamilton BB, Sherwin FS (1987). The functional independence measure: a new tool for rehabilitation. Adv Clin Rehabil.

[12] Sutherland JM, Walker J (2008). Challenges of rehabilitation case mix measurement in Ontario hospitals. Health Policy.

[13] Hall RE, Sondergaard D, Wodchis WP, Fang J, Mondal P, Bayley MT (2017). Trajectories of stroke Care in Ontario: which path to best care?. Can J Neurol Sci..

[14] Hebert D, Lindsay MP, McIntyre A (2016). Canadian stroke best practice recommendations: stroke rehabilitation practice guidelines, update 2015. Int J Stroke.

[15] Allen L, Richardson M, McIntyre A (2014). Community stroke rehabilitation teams: providing home-based stroke rehabilitation in Ontario, Canada. Can J Neurol Sci.

[16] Rice D, Janzen S, McIntyre A, Vermeer J, Britt E, Teasell R (2016). Comprehensive outpatient rehabilitation program: hospital-based stroke outpatient rehabilitation. J Stroke Cerebrovasc Dis.

[17] Centers for Disease Control and Prevention (CDC) (2007). Outpatient rehabilitation among stroke survivors--21 states and the District of Columbia, 2005. MMWR Morb Mortal Wkly Rep.

[18] Sandel M, Wang H, Terdiman J (2009). Disparities in stroke rehabilitation: results of a study in an integrated health system in northern California. PMR..

[19] Chan L, Wang H, Terdiman J (2009). Disparities in outpatient and home health service utilization following stroke: results of a 9-year cohort study in northern California. PMR..

[20] Freburger J, Holmes G, Ku L, Cutchin M, Heatwole-Shank K, Edwards L (2011). Disparities in post-acute rehabilitation Care for Stroke. Arch Phys Med Rehabil.

[21] Burton JK, Ferguson EEC, Barugh AJ (2018). Predicting discharge to institutional long-term care after stroke: a systematic review and Metaanalysis. J Am Geriatr Soc.

[22] Brown AW, Therneau TM, Schultz BA, Niewczyk PM, Granger CV (2015). Measure of functional independence dominates discharge outcome prediction after inpatient rehabilitation for stroke. Stroke..

[23] Johnson J, Smith G, Wilkinson A (2015). Factors that influence the decision-making of an interdisciplinary rehabilitation team when choosing a discharge destination for stroke survivors. Can J Neurosci Nurs.

[24] Mirkowski M, Pereira S, Janzen S (2018). Caregiver availability for severe stroke results in improved functional ability at discharge from inpatient rehabilitation. Disabil Rehabil.

[25] Chen L, Xiao LD, De Bellis A (2016). First-time stroke survivors and caregivers' perceptions of being engaged in rehabilitation. J Adv Nurs.

[26] Weiss Z, Snir D, Zohar R (2004). Allocation and preference of patients for domiciliary or institutional rehabilitation after a stroke. Int J Rehabil Res.

[27] Godwin K, Wasserman J, Ostwald S (2011). Cost associated with stroke: outpatient rehabilitative services and medication. Top Stroke Rehabil.

[28] Grace SL, McDonald J, Fishman D, Caruso V (2005). Patient preferences for home-based versus hospital-based cardiac rehabilitation. J Cardpulm Rehabil.

[29] Landry MD, Jaglal S, Wodchis WP, Raman J, Cott CA (2008). Analysis of factors affecting demand for rehabilitation services in Ontario, Canada: a health-policy perspective. Disabil Rehabil.

